# Molecular epidemiology of malaria parasite amongst patients in a displaced people’s camp in Sudan

**DOI:** 10.1186/s41182-020-0192-3

**Published:** 2020-01-29

**Authors:** Hamza Adam Eshag, Elfadel Elnzer, Elkhatieb Nahied, Mustafa Talib, Ali Mussa, Abd Elhafiz M. A. Muhajir, Ibrahim Khider Ibrahim, Abdulwali Sabo, Salah-Eldin Gumma Elzaki, Zeehaida Mohamed, Khalid Hajissa

**Affiliations:** 1 Department of Zoology, Faculty of Science and Technology, Omdurman Islamic University, B.O.Box 382, Omdurman, Sudan; 2 Department of Haematology, Faculty of Medical Laboratory Sciences, Al Neelain University, Khartoum, Sudan; 3 Unit of Biostatistics and Research Methodology, School of Medical Sciences, Universiti Sains Malaysia, Health Campus, 16150 Kubang Kerian, Kelantan Malaysia; 4 Department of Molecular Epidemiology, Tropical Medicine Research Institute, National Center for Research, Khartoum, Sudan; 5 Department of Medical Microbiology & Parasitology, School of Medical Sciences, Universiti Sains Malaysia, 16150 Kubang Kerian, Kelantan Malaysia

**Keywords:** Malaria, *Plasmodium*, Molecular epidemiology, Displaced camp, Sudan, Nested PCR

## Abstract

**Background:**

Despite the importance of epidemiological studies in the development of effective control strategies and provision of basic health services for refugees and internally displaced persons (IDPs), data on the prevalence of malaria are limited. Thus, this study was conducted to estimate the molecular prevalence of malaria amongst the displaced population in Ardamata IDP camp in Al-Geneina City, Sudan.

**Methods:**

A cross-sectional study was conducted from July 2018 to December 2018 to estimate malaria prevalence amongst the displaced population in Ardamata IDP camp in Al-Geneina City, Sudan. A total of 380 patients with suspected malaria were recruited. Nested polymerase chain reaction (nPCR) assays were performed to detect the *Plasmodium* genus and species.

**Results:**

Of 380 patients, 232 (61.1%) were positive for malaria. *Plasmodium falciparum* was the only prevalent species detected amongst the study population. nPCR analysis revealed that none of the samples had *Plasmodium vivax*, *Plasmodium ovale* or *Plasmodium malariae*. The malaria prevalence rate was higher amongst males (67.1%) than in females (56.8%), and gender was the only risk factor that was significantly associated with malaria infection (*p =* .042).

**Conclusions:**

Despite control programmes, malaria remains a significant cause of illness amongst a displaced population. The high prevalence of malaria infection in this study indicates that additional health facilities and control strategies should be implemented in displaced camps and the surrounding areas.

## Background

Malaria is a fatal vector-borne tropical disease that remains one of the leading causes of death in many developing countries [1]. The disease continues to pose global public health challenges, and its related morbidity and mortality remain significantly high in endemic countries such as Sudan. Although intensive control measures in recent years have resulted in a substantial reduction in the disease burden, the limited control options and availability of resources due to the violent conflict in Darfur maintain the high risk of malaria in displaced camps; vulnerability to malaria might be promoted by many factors including decimated health care infrastructure and social disruption, making the disease responsible for most cases of death [2]. The high prevalence of malaria in displaced populations in Africa constitutes an emerging challenge for humanitarian response as the disease becomes a serious health problem amongst internally displaced persons (IDPs) in these areas. In Sudan, malaria is one of the most concerning infectious diseases amongst displaced populations, and data on malaria prevalence in displaced camps are extremely limited. Additional epidemiological information is required for the development of effective control strategies and provision of basic health services, because the overall aim of any epidemiological study is to prevent and reduce excess mortality and morbidity. According to the World Health Organisation, five *Plasmodium* species have been recognised as the causative agents of malaria that can infect humans: *Plasmodium falciparum* (*P*. *falciparum*), *Plasmodium vivax* (*P*. *vivax*), *Plasmodium ovale* (*P*. *ovale*), *Plasmodium malariae* (*P*. *malariae*) and *Plasmodium knowlesi* (*P*. *knowlesi*). Of these five species, *P*. *falciparum* and *P*. *vivax* are the most common in Sudan.

Blood film microscopy and rapid diagnostic tests are the mainstay of malaria diagnosis that can adequately detect *Plasmodium* infections in patients with high levels of parasitaemia [3, 4]. However, both methods lack the sensitivity to detect the infection in individuals carrying low parasite density [5, 6]. Given that low-grade parasitaemia in asymptomatic individuals can persist for a year or more, important sources of further transmission must be considered. Recent reports showed that sub-microscopic infections represent about 70–80% of all *Plasmodium* infections amongst children, pregnant women and non-pregnant adults [7]. Thus, highly sensitive diagnostic methods are necessary. In recent years, several molecular methods have been developed and evaluated for the detection of *Plasmodium* species, and various sensitivities and specificities have been reported [1]. Amongst them, polymerase chain reaction (PCR) is the most frequently used method in the field [8]. PCR has also been helpful in the differential detection of all malaria parasites up to species levels, thereby revealing the high prevalence of mixed infections [1, 9]. The application of sensitive methods such as PCR to determine the prevalence of *Plasmodium* species will allow better documentation of malaria epidemiology [10] and overcome the lack of knowledge on the prevalence of malaria infection in the displaced population. This study was proposed to determine the molecular prevalence of malaria parasites amongst symptomatic patients in Ardamata IDP camp, Al-Geneina City, Sudan.

## Methods

### Study setting

This study was carried out in Ardamata IDP camp established in Al-Geneina City, which is the capital city of West Darfur State, the western part of Sudan. It is located in the latitude of 13° 27′ 15′′ and longitude of 22° 26′ 8′′. Al-Geneina is approximately 1200 km from the capital city of Khartoum.

### Study design and population

This study was a cross-sectional study that recruited patients presenting clinical symptoms of malaria and visiting the health centre in the study site. A total of 380 patients were recruited for this study between July and December 2018.

### Samples size calculation

The sample size was estimated using formula for single proportion to estimate the prevalence of malaria.

$$ \left(\frac{z}{m}\right)2\ \mathrm{x}\ \mathrm{p}\ \left(1-\mathrm{p}\right) $$

The parameters used were *z* = 1.96 (for 0.05 level of significance), margin of error (m) = 0.05, *p* = 0.575 and 0.33 for *P*. *falciparum* and *P*. *vivax* respectively [11]. The calculated sample size was 376 and 340 for *P*. *falciparum* and *P*. *vivax* respectively. Hence, the largest sample size based on *P*. *falciparum* (376) was used. After adding 5% dropout rate, the adjusted samples size was estimated as 396.

### Sample collection

About 3–5 drops of blood from each enrolled participant were collected on Whatman No. 1 filter paper. The blood samples were allowed to dry, kept in individual plastic bags with desiccant and stored at room temperature. An informed consent questionnaire was used to collect individual socio-demographic data.

### DNA extraction

DNA was extracted from three 3 mm punches of dried blood spot (DBS) following the protocol of Bereczky et al. [12]. DNA was eluted in a total volume of 50 μl of tri-EDTA buffer (TE) buffer and stored at − 20 °C.

### PCR for *Plasmodium* detection

Nested PCR (nPCR) was performed as described previously [13] in a two-step procedure. In the first PCR round, amplification was performed using rPLU1 and rPLU5 primers for *Plasmodium* genus determination. The PCR mixture was prepared in a total volume of 20 μl, containing 10 μl of MyTaq™ mix (Bioline, UK), 0.4 μM of each primer and 2.5 μl of extracted DNA. PCR was performed under the following conditions: 94 °C for 5 min as the initial denaturation step; 25 cycles at 94 °C for 45 s, 58 °C for 45 s and 72 °C for 1 min; and a final extension step at 72 °C for 5 min. The amplified PCR product (1 μl) was used as a template for the second PCR round for *Plasmodium* species identification using (rFAL1 and rFAL2, rOVA-1 and rOVA2, rVIV1 and rVIV2, rMAL1 and rMAL2) primers [13]. The reaction mix contained 10 μl of MyTaq™ mix (Bioline, UK) and 0.4 μM of each primer, and the final reaction volume was made up to 20 μl by adding double distilled water. Amplification was performed under the following conditions: 95 °C for 5 min; 30 cycles of 94 °C for 1 min, 58 °C for 2 min and 72 °C for 5 min; and final extension at 72 °C for 2 min. A known *Plasmodium* positive samples and a negative control sample without DNA template was used in all the reactions as positive and negative control respectively.

### Data analysis

Preliminary data analysis was conducted for data exploration and cleaning to check for missing values and erroneous data entry. The statistical analysis applied in the current study was descriptive analysis and logistic regression analysis. Descriptive analysis focused on frequency, percentages, mean and standard deviation. Logistic regression analysis was conducted to identify significant factors associated with the outcome of malaria infection. All statistical analyses were conducted using SPSS 24.

## Results

### Socio-demographic characteristics of the study participants

Table 1 shows the descriptive characteristics of the participants. A total of 380 patients with clinical suspicion of malaria were enrolled in this study. Approximately 96.1% (*n* = 365) of the participants were residents of Ardamata IDP camp. Amongst the study participants, 41.6% (*n* = 158) were males and 58.4% (*n* = 222) were females. Their ages ranged from 1 to 80 years, and the mean age was 21.7 years (SD = 14.1). The majority of the participants (59.5%, *n* = 226) belonged to the < 20 age group. More than half of the participants were single (58.9%, *n* = 224). Majority of them (44.5%, *n* = 169) reported receiving primary school education, whereas 34.7% (*n* = 132) were illiterate. The monthly income was < 20 USD for 14.5% (*n* = 55), 20–25 USD for 46.6% (*n* = 177), 25–30 USD for 20% (*n* = 76) and more than 30 USD for 18.9% (*n* = 72). Approximately 97.9% (*n* = 372) of the patients with suspected malaria had mosquito nets.

**Table 1:** General and socio-demographic characteristics of participants

Variables	Categories	Frequency	Percentage	Mean (SD)
Residence	Resident	365	96.1	
	Visitor	15	3.9	
Gender	Male	158	41.6	
	Female	222	58.4	
Age				21.7 (14.1)
Occupation	None (student and retired)	252	66.3	
	Self-employed (farming and other)	114	30	
	Gov. employee	14	3.7	
Marital status	Married	156	41.1	
	Single	224	58.9	
Monthly income	< 20 USD	55	14.5	
	20–25 USD	177	46.6	
	25–30 USD	76	20	
	> 30 USD	72	18.9	
Education level	Illiterate	132	34.7	
	Primary	169	44.5	
	Secondary	59	15.5	
	Graduate and above	20	5.3	
Fever	No	69	18.2	
	Yes	311	82.8	
Joint pain	No	256	67.4	
	Yes	124	32.6	
Mosquito net	No	8	2.1	
	Yes	372	97.9	
Malaria PCR	Negative	148	38.9	
	Positive	232	61.1	

### Prevalence of malaria infection and the associated risk factors

**Fig. 1:**
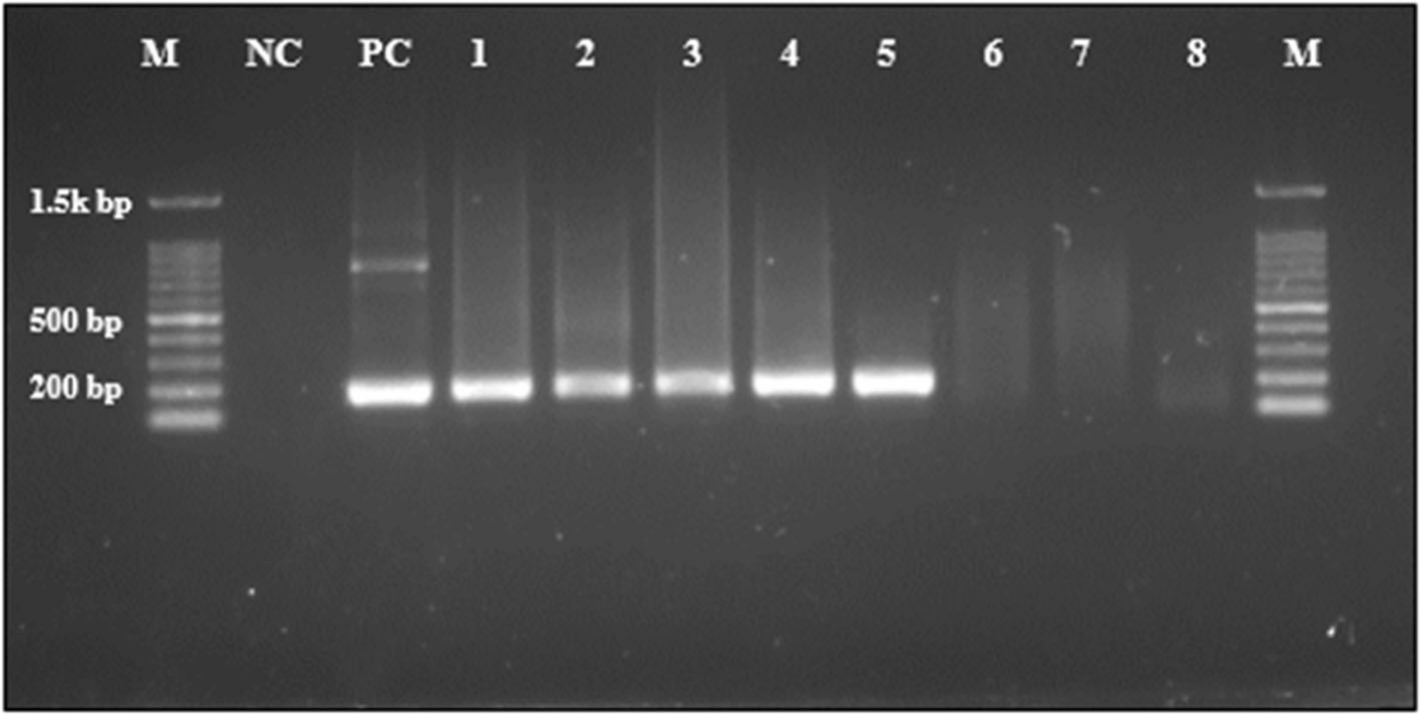
DNA amplification of *Plasmodium* species by nPCR. Lane M: 100 bp DNA Marker. Lane NC: negative control. Lane PC: positive control. Lane 1–5: positive samples for *P. falciparum* t. Lane 6–8: negative control

Molecular analysis showed that approximately 61.1% (232 out of 380) of analysed samples were positive for malaria (Table 1). *P*. *falciparum* was the only prevalent species found amongst the study population (Fig. 1). None of the samples had *P*. *vivax*, *P*. *ovale* or *P*. *malariae*. The prevalence of malaria infection was higher in males (67.1%) than in females (56.8%). Gender had a statistically significant association with malaria infection (crude odds ratio [COR] = 1.55, *p* = .042), indicating that the males were 1.5 times more likely to have malaria infection than the females. None of the remaining factors demonstrated any significant association with malaria infection (Table 2).). For example, the unadjusted crude odds ratio of age indicated that those who are 21 years and above were 11% less likely to have malaria infection than the 20 years and below group (COR = 0.89, *p* value = 0.579).

**Table 2:** Factors associated with test positivity for malaria

Characteristics	Test positivity				
	Total No. (%)	Negative No. (%)	Positive No. (%)	Crude OR (95% CI)	*P* value
Residence					
Resident	365 (96.1)	142 (38.9)	223 (61.1)	1.047 (0.37, 3.00)	.932
Visitor	15 (3.9)	6 (40.6)	9 (60.0)	1	
Gender					
Male	158 (41.6)	52 (32.9)	106 (67.1)	1.55 (1.02, 2.38)	.042
Female	222 (58.4)	96 (43.2)	126 (56.8)	1	
Age group					
≤ 20 years	230 (60.5)	87 (37.8)	143 (62.2)	1	
≥ 21 years	150 (39.5)	61 (40.7)	89 (59.3)	0.89 (0.58, 1.35)	0.579
Occupation					
None (student and retired)	252 (66.3)	101 (40.1)	151 (59.9)	1	
Self-employed (farming and other)	114 (30.0)	41 (36.0)	73 (64.0)	1.19 (0.75, 1.88)	0.445
Gov. employee	14 (3.7)	6 (42.9)	8 (57.1)	0.89 (0.30, 2.65)	.837
Monthly income					
< 20 USD	55 (14.5)	18 (32.7)	37 (67.3)	1	
20–25 USD	177 (46.6)	75 (42.4)	102 (57.6)	0.66 (0.35, 1.25)	.204
25–30 USD	76 (20)	23 (30.3)	53 (69.7)	1.12 (0.53, 2.36)	.764
> 30 USD	72 (18.9)	32 (44.4)	40 (55.6)	0.61 (0.29, 1.26)	.182
Marital status					
Married	156 (41.1)	60 (38.5)	96 (61.5)	1	
Single	224 (58.9)	88 (39.3)	136 (60.7)	0.97 (0.64,1.47)	.871
Education level					
Illiterate	132 (34.7)	49 (37.1)	83 (62.9)	1	
Primary	169 (44.5)	69 (40.8)	100 (59.2)	0.86 (0.54, 1.37)	.513
Secondary	59 (15.5)	23 (39.0)	36 (61.0)	0.92 (0.49, 1.730	.806
Graduate and above	20 (5.3)	7 (35.0)	13 (65.0)	1.10 (0.41, 2.93)	.855
Fever					
No	69 (18.2)	29 (42.0)	40 (58.0)	1	
Yes	311 (81.8)	119 (38.3)	192 (61.7)	1.170	.562
Joint pain					
No	256 (67.4)	94 (36.7)	162 (63.3)	1	
Yes	124 (32.6)	54 (43.5)	70 (56.5)	.752	.201

*COR* crude odds ratio, *AOR* adjusted odds ratio, *CI* confidence interval, *SD* standard deviation

## Discussion

Malaria remains one of the significant health problems in the tropical and subtropical poorest nations [14]. In Sudan, the disease is endemic, and previous studies reported a relatively high burden of the disease in many areas of the country. In this study, blood samples were collected from patients suspected to have malaria at Ardamata IDP camp. Genus- and species-specific nPCR was used as a diagnostic tool to detect the malaria parasites. A high prevalence of the malaria parasites (61.1%) was detected amongst the study participants. This percentage was remarkably high compared with the low prevalence of malaria infection detected by microscope in Dar Alsalam (5%) and Jabal Awlia (11%) camps [15], which are located in Khartoum state. This low infection rate could be attributed to the prevention and control activities of malaria in these areas. However, when a similar diagnostic method was used (nPCR), high prevalence of *Plasmodium* parasites was also detected amongst patients with suspected malaria recruited from different clinics in Omdurman area [16] and Kosti [17] (44.1% and 32%), respectively. The significantly high burden of malaria infection in this study may have coincided with the limited control options and availability of resources in the displaced camp. It may also be a result of the timing of sample collection due to the malaria transmission season.

Similarly, the discrepancy in disease epidemiology was also reported in other African countries. A study conducted in Ethiopia, a malaria-endemic country, revealed that the overall prevalence rate of malaria detected by microscopy was 18.4% [18]. The molecular detection of malaria parasite in Democratic Republic of Congo and Nigeria demonstrated that the infection rate of the disease was 34.9% [8] and 58.7% respectively [10].

In endemic areas, determination of the epidemiological pattern of the malaria infection is crucial for intervention programmes and treatment purposes. Accordingly, all the main malaria species that infect humans have been previously reported in the country [19], with a predominance of *P*. *falciparum* malaria and relatively rare *P*. *vivax* infection in regions of the study. However, most of the recent studies indicated changes in the epidemiological pattern, and a high proportion of *P*. *vivax* infections was reported [20]. The results of the current study revealed a high infection rate of *P*. *falciparum* malaria. This elevated prevalence amongst the overall study population has also been reported by previous research [21]. None of the samples of the present study were positive for *P*. *vivax*, *P*. *ovale* and *P*. *malariae*. However, many previous studies have demonstrated the presence of non-*P*. *falciparum* elsewhere in the country, either through single or mixed infections. For instance, out of 283 malaria-positive cases, Ageep (2013) reported that 50.2% was *P*. *falciparum*, 43.8% was *P*. *vivax*, 04.9% was *P*. *ovale* and 1.1% of the cases was *P*. *malariae*; no mixed infection was observed [19]. Recently, a remarkable increase in the recurrent relapses of malaria infections was observed in different areas in Sudan, thereby indicating a high infection rate of *P*. *vivax* malaria and making *P*. *vivax* the second important species in the country [11]. Another recent study showed that the occurrence of *P*. *vivax* malaria is high amongst suspected malaria cases, with a prevalence of 26.6% [20]. In general, the variation in the overall prevalence and species-specific malaria might be due to differences in the geography of the study area, sample size used, timing of sample collection, climate condition, study subjects, environmental factors and many other factors involved [22].

Univariate regression analysis of the risk of having malaria in suspected symptomatic participants showed that only gender was significantly associated with malaria (COR = 1.55, *p* = .042), and none of the remaining factors had any significant influence (Table 2).

The prevalence of malaria infection in relation to gender indicated that the males were 1.5% more likely to have malaria infection than the females. The higher prevalence observed amongst males in this study was in agreement with the findings of previous reports that showed predominance of malaria infection in males [23, 24] but contradicted other studies [25, 26]. Some hypotheses justify the increased infection rate amongst males by the fact that they are more likely to work outside compared with females; thus, men are subjected to an increased number of infected mosquito bites than females [27].

In malaria-endemic areas, protective immunity is always correlated with age. A low prevalence of malaria and low incidence of clinical symptoms are frequently observed amongst adults and older children. This concept is in line with the observations of this study, which showed that the odds of being positive for malaria decreased by 11% amongst those who are ≥ 21 years compared to the ≤ 20 years (*p* value = 0.579). No associations were found in the present study between malaria infection and the use of insecticide-treated bed net (ITNs). Similarly, education status and marital status did not show any significant association with malaria infection.

These findings were in contrast to other work, which showed that the use of ITNs and many socio-demographic factors are significantly associated with malaria. Recruiting only patients with suspected clinically symptomatic malaria possibly affected the results of this study. Further comprehensive surveys are required to identify the factors associated with malaria infection that were not addressed in this study.

## Conclusion

In conclusion, results of this study indicated a high prevalence of malaria amongst the displaced participants. This study further emphasises the necessity to strengthen malaria control strategies and establish additional health facilities.

## Data Availability

Any further requested information regarding the experimental and data analysis during the current study is available from the corresponding author on reasonable request.

## Abbreviations

DBS: Dried blood spot
IDPs: Internally displaced persons
ITN: Insecticide-treated bed net
nPCR: Nested polymerase chain reaction
TE: Tri-EDTA buffer
